# Supplementary material for: A Biomarker Panel Based upon AFP, Fucosylated Kininogen and PEG-Precipitated IgG Is Highly Accurate for the Early Detection Hepatocellular Carcinoma in Patients with Cirrhosis in Phase II and Phase III Biomarker Evaluation
Source: Cancers (Basel). 2022 Dec 2;14(23):5970. doi: 10.3390/cancers14235970 (PMC9740205; doi:10.3390/cancers14235970)
Supplement: Supplementary file 1 [file cancers-14-05970-s001.zip › cancers-1974393-supplementary.pdf]

S1

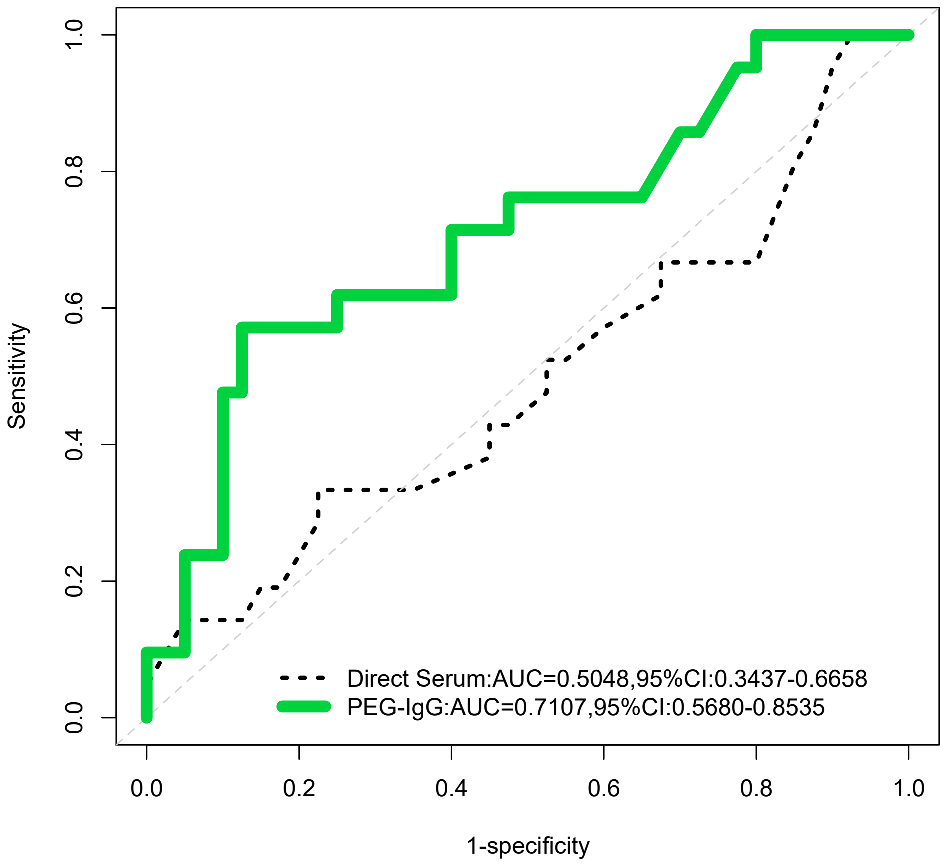

Supplementary Figure S1: Peg precipitation required for Biomarker performance. IgG was analyzed in 40 cirrhotic or 21 early stage HCC samples (same as in Figure 1) either by total IgG analysis or after PEG precipitation. Without PEG precipitation, there was no discriminatory ability to differentiate the HCC from the cirrhotic group (AUC = 0.5048, $p = 0.9516$). The AUC of the PEG-IgG material was statistically different than the AUC of total IgG (Delong's test, $p < 0.001$).

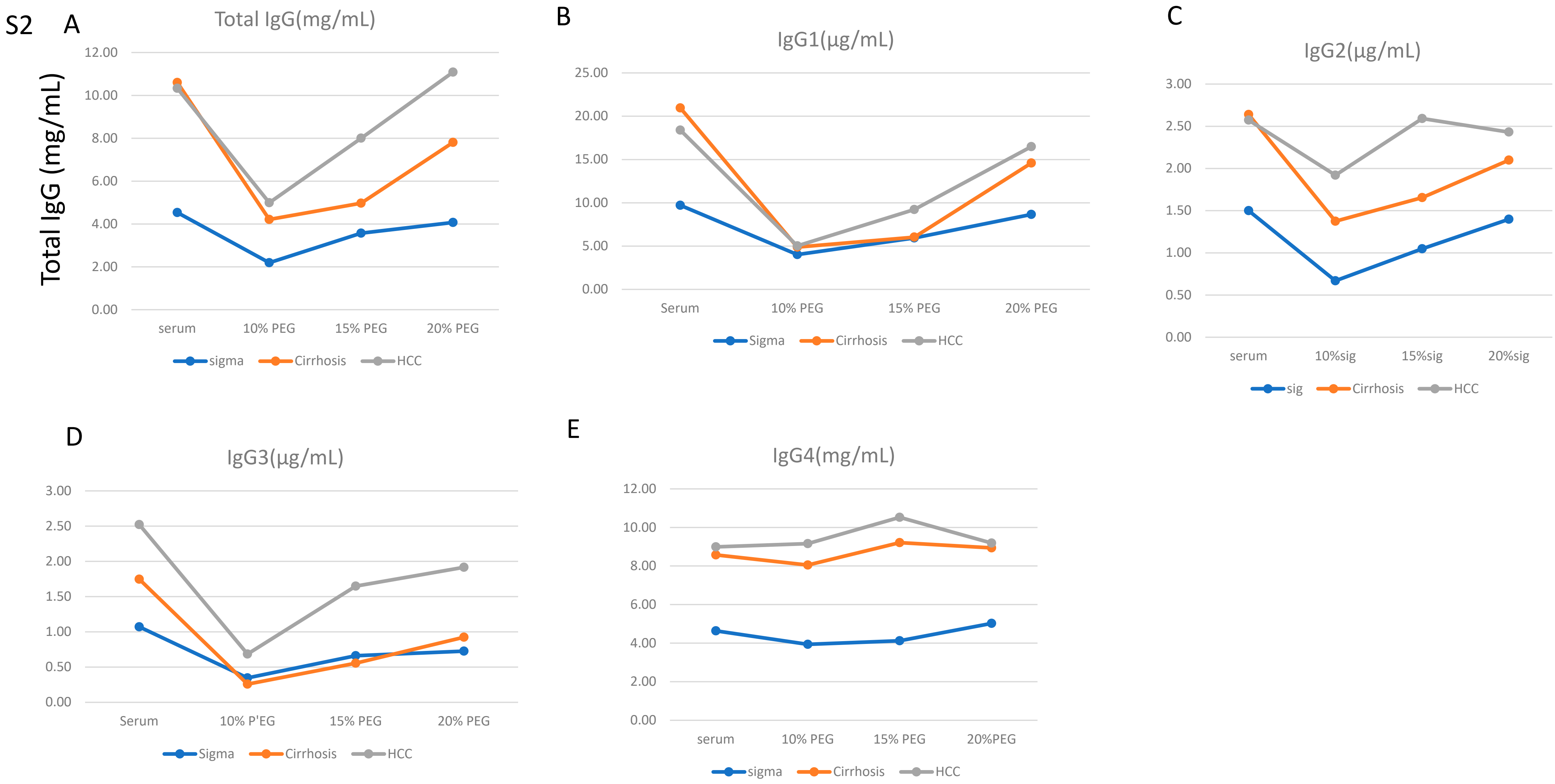

Supplementary Figure S2: Examination of the IgG subtypes in associated with PEG-IgG biomarker performance. A pool of HCC (22 samples), cirrhotic (22 samples), and commercially purchased "healthy" serum for subtype analysis. These three serum samples were precipitated in 10%, 15% or 20% PEG, and the pellets were quantified for their IgG subtypes using a commercially available human IgG subtyping kit. (A) total IgG detection' (B) IgG1 detection; (C) IgG2 detection, (D) IgG3 detection and (E) IgG4 detection.

S3

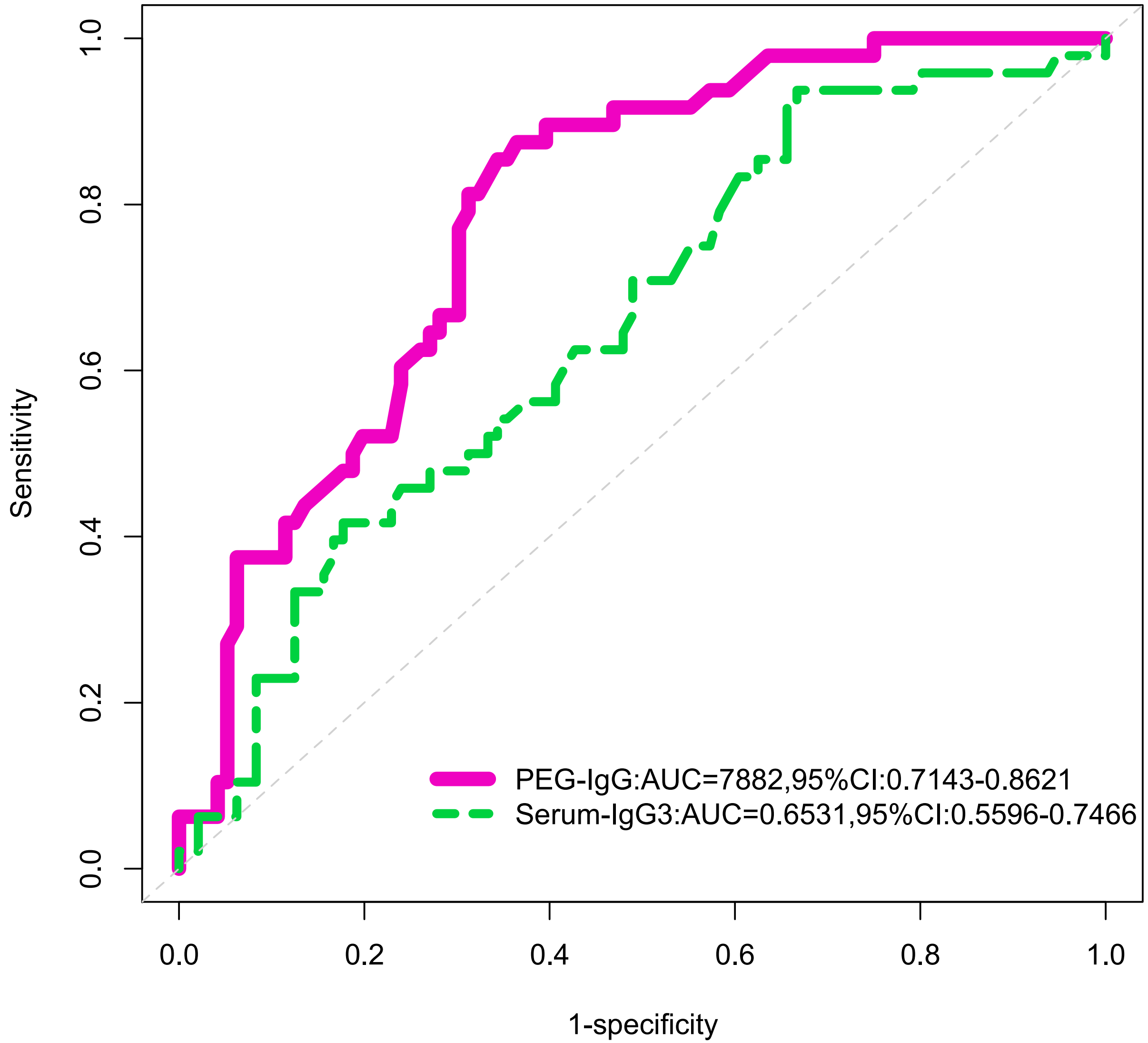

Supplementary Figure S3: AUROC for either total IgG (following PEG precipitation) or for IgG3 (following PEG precipitation) from the 22 cirrhotic and 22 HCC samples (a subset for the UTSW samples in Table 1).

S4

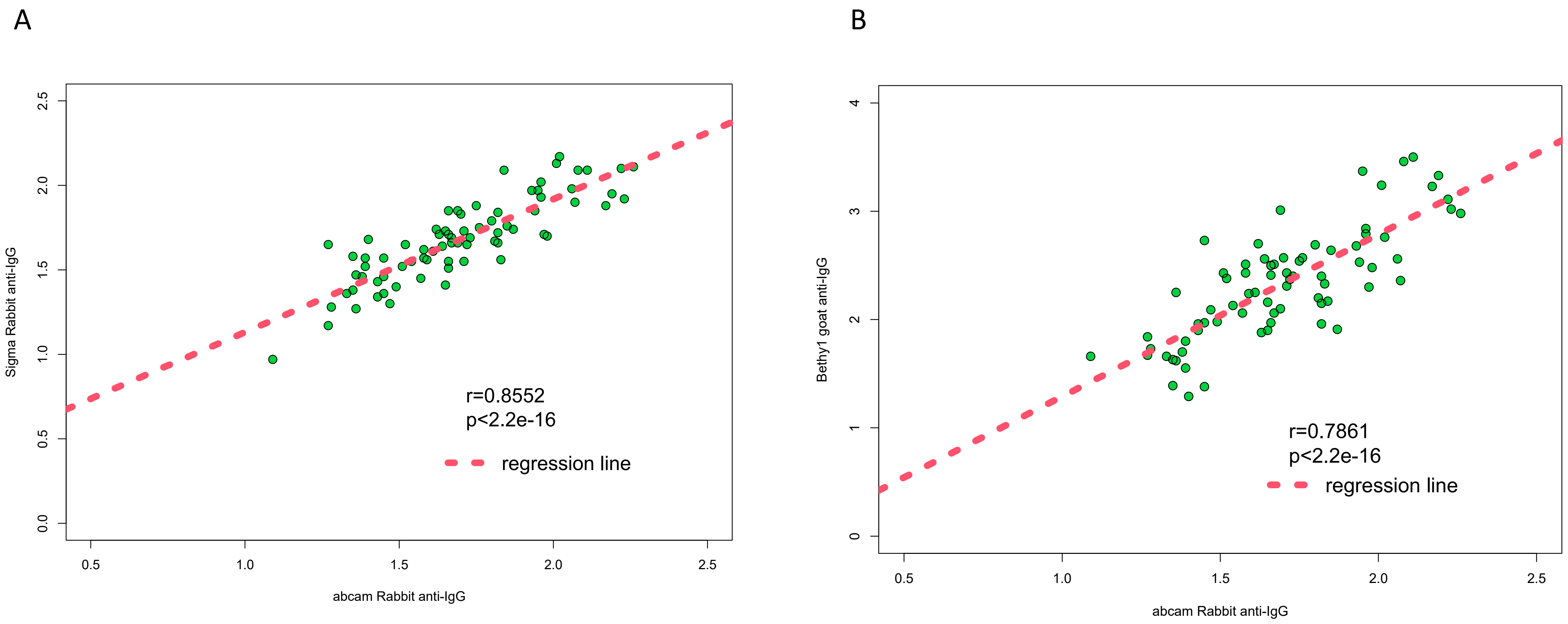

Supplementary Figure S4: Similar performance of multiple IgG antibodies. (A) Comparison of Sigma anti-IgG with Abcam anti-IgG. (B) Comparison between Bethyl anti-IgG and Abcam anti-IgG.

S5

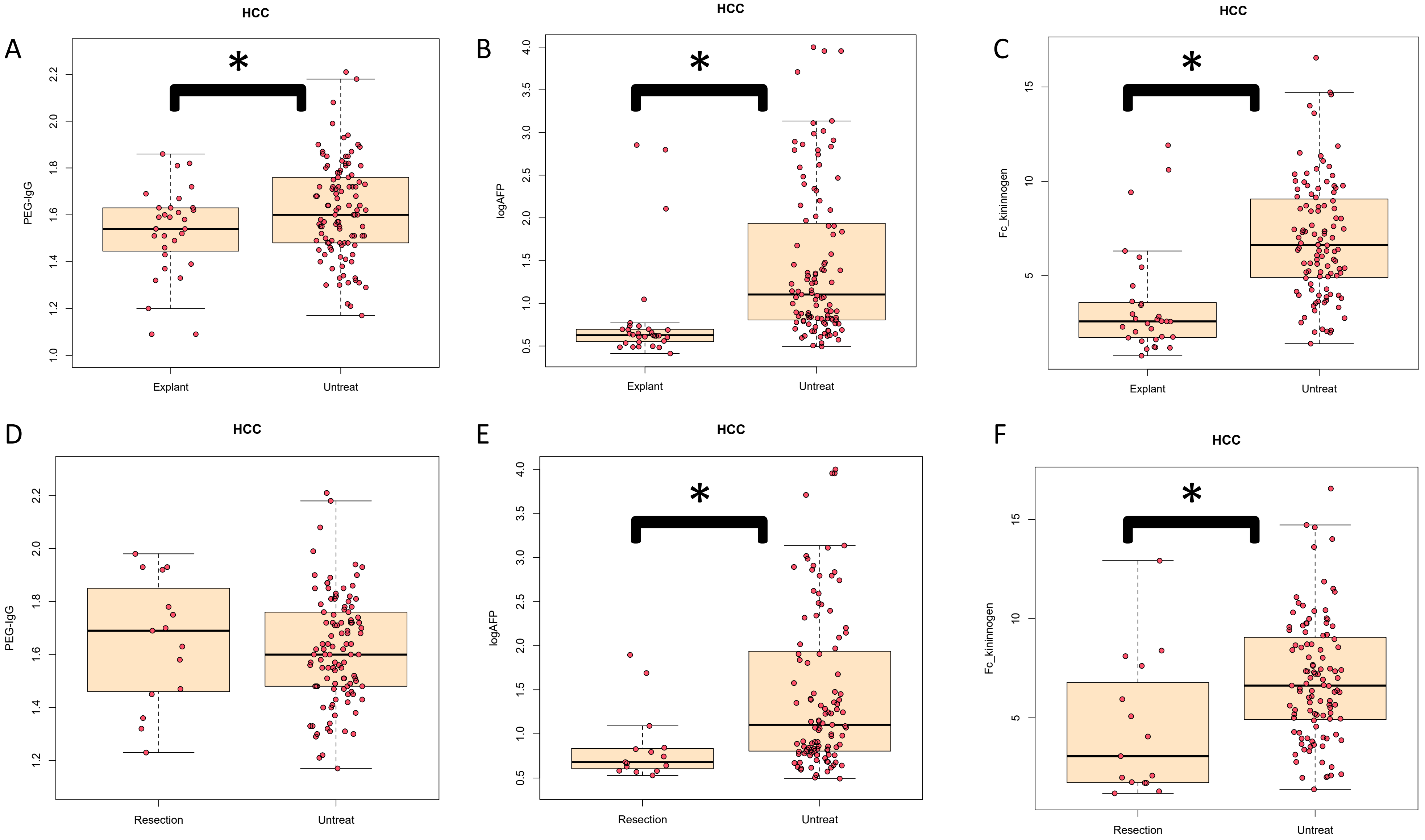

Supplementary Figure S5: Performance of biomarkers in patients treated for the HCC. Peg-IgG (A), AFP (B) or fucosylated kininogen (C) in patients either following transplant (explant) or untreated. Peg-IgG (D), AFP (E) or fucosylated kininogen in patient whose tumors were resected or untreated. Asterisk indicated statistical difference (Mann Whitney, $p < 0.05$)

S6

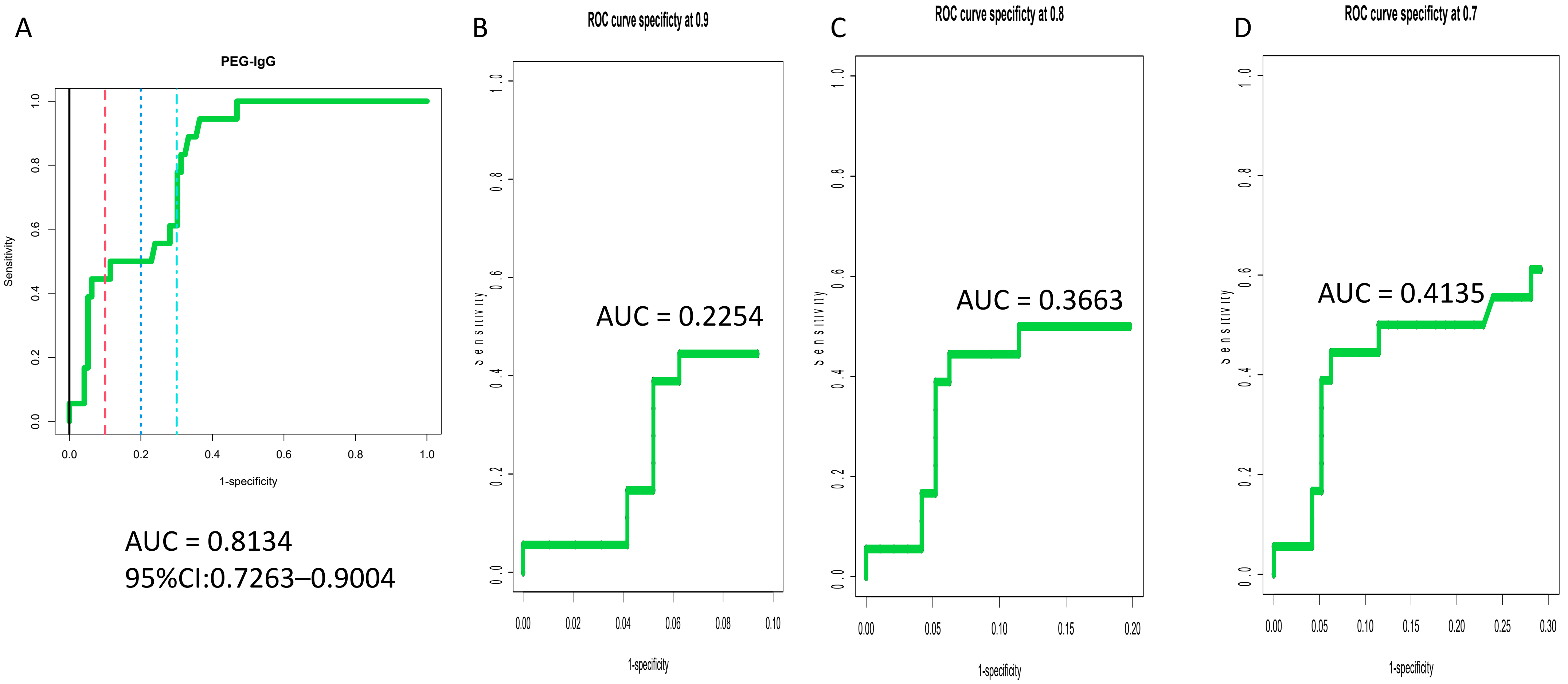

Supplementary Figure S6: AUROC of PEG-IgG in the UM cohort in patients at a time point of 1–6 months prior to HCC development. (A) Full ROC of Peg-IgG showing the specificity cut-offs at 90% specificity, 80% specificity and 70% specificity. Partial ROCS at 90% (B), 80% (C) or 70% specificity (D) are shown with the AUC for each partial ROC provided.

S7

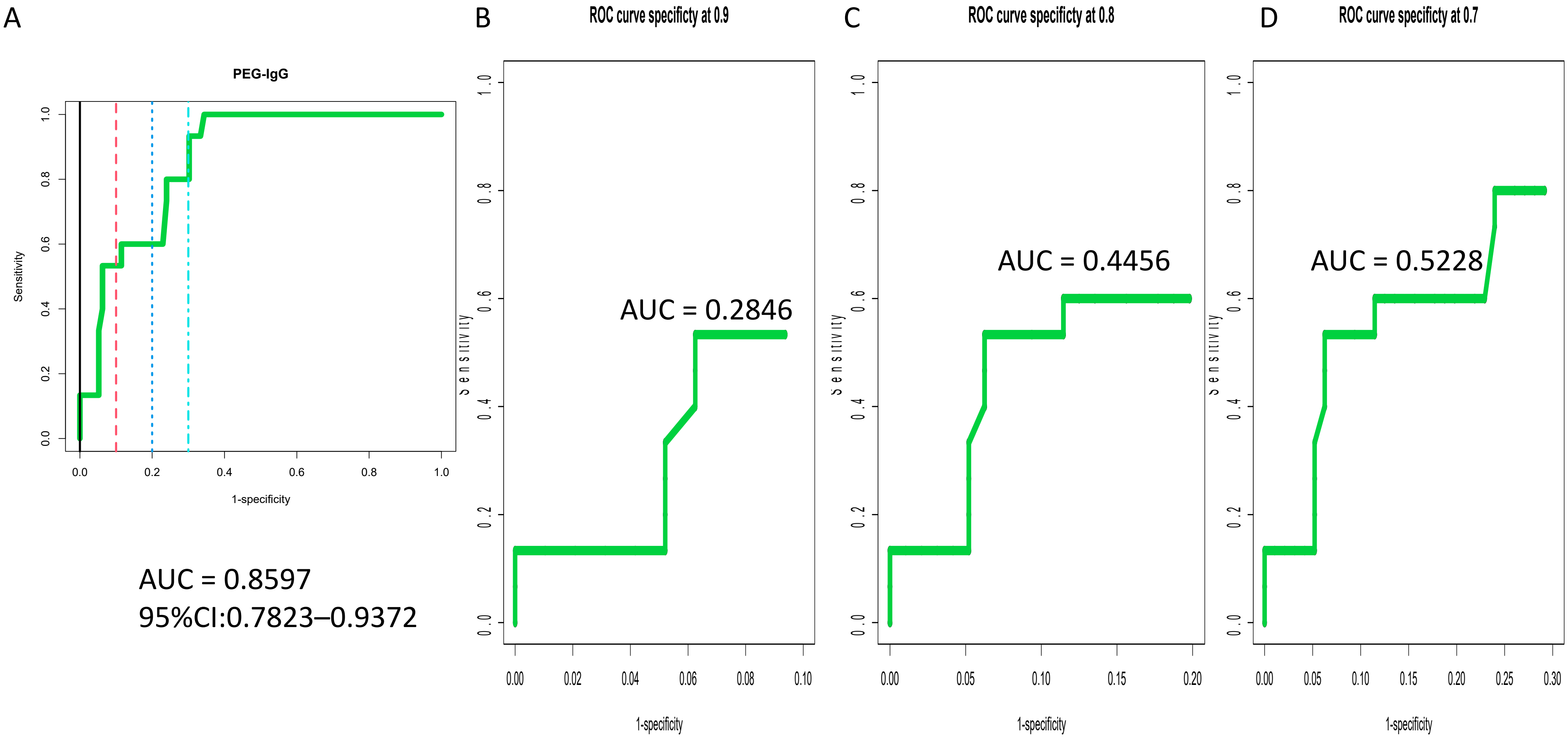

Supplementary Figure S7: AUROC of PEG-IgG in the UM cohort in patients at a time point of 7–12 months prior to HCC development. (A) Full ROC of Peg-IgG showing the specificity cut-offs at 90% specificity, 80% specificity and 70% specificity. Partial ROCS at 90% (B), 80% (C) or 70% specificity (D) are shown with the AUC for each partial ROC provided.

S8

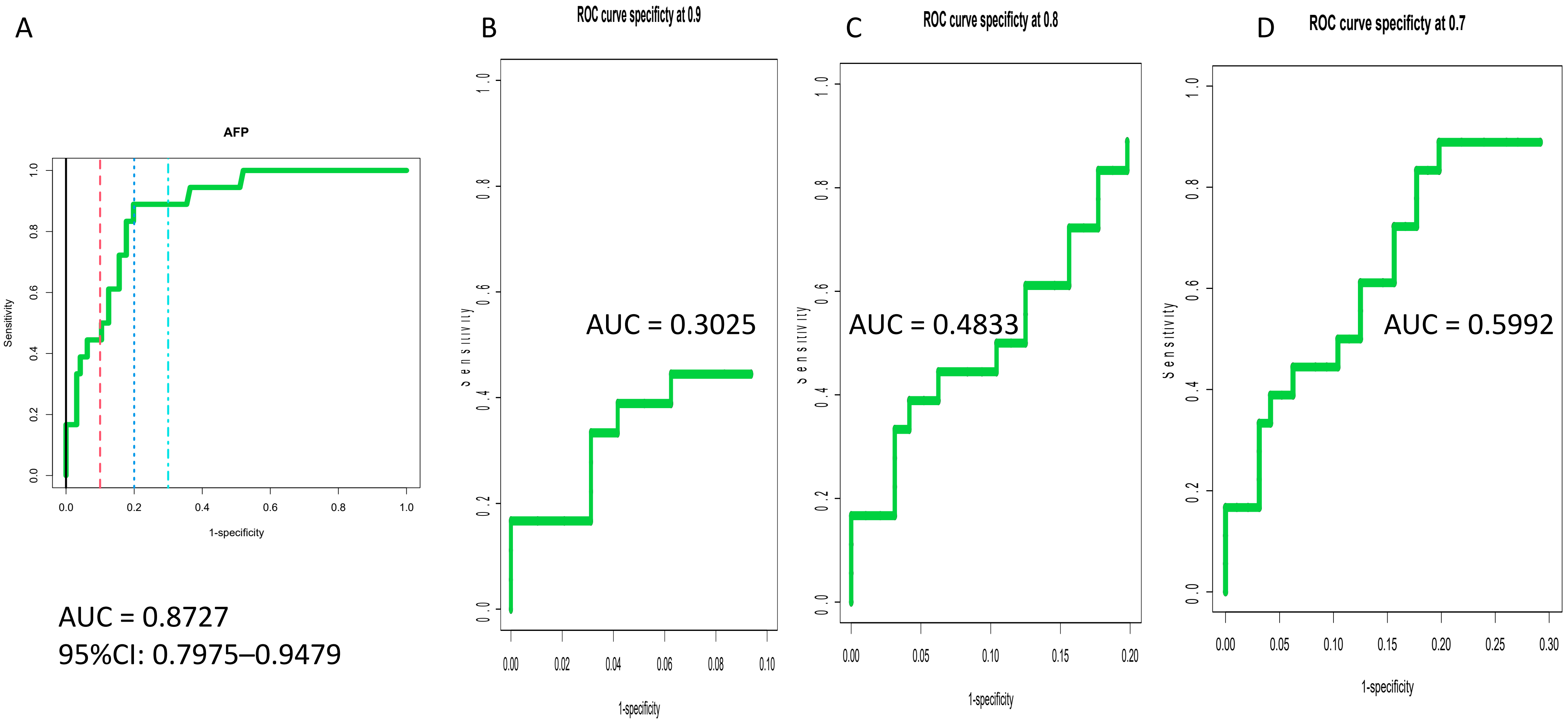

Supplementary Figure S8: AUROC of AFP in the UM cohort in patients at a time point of 1–6 months prior to HCC development. (A) Full ROC of Peg-IgG showing the specificity cut-offs at 90% specificity, 80% specificity and 70% specificity. Partial ROCS at 90% (B), 80% (C) or 70% specificity (D) are shown with the AUC for each partial ROC provided.

S9

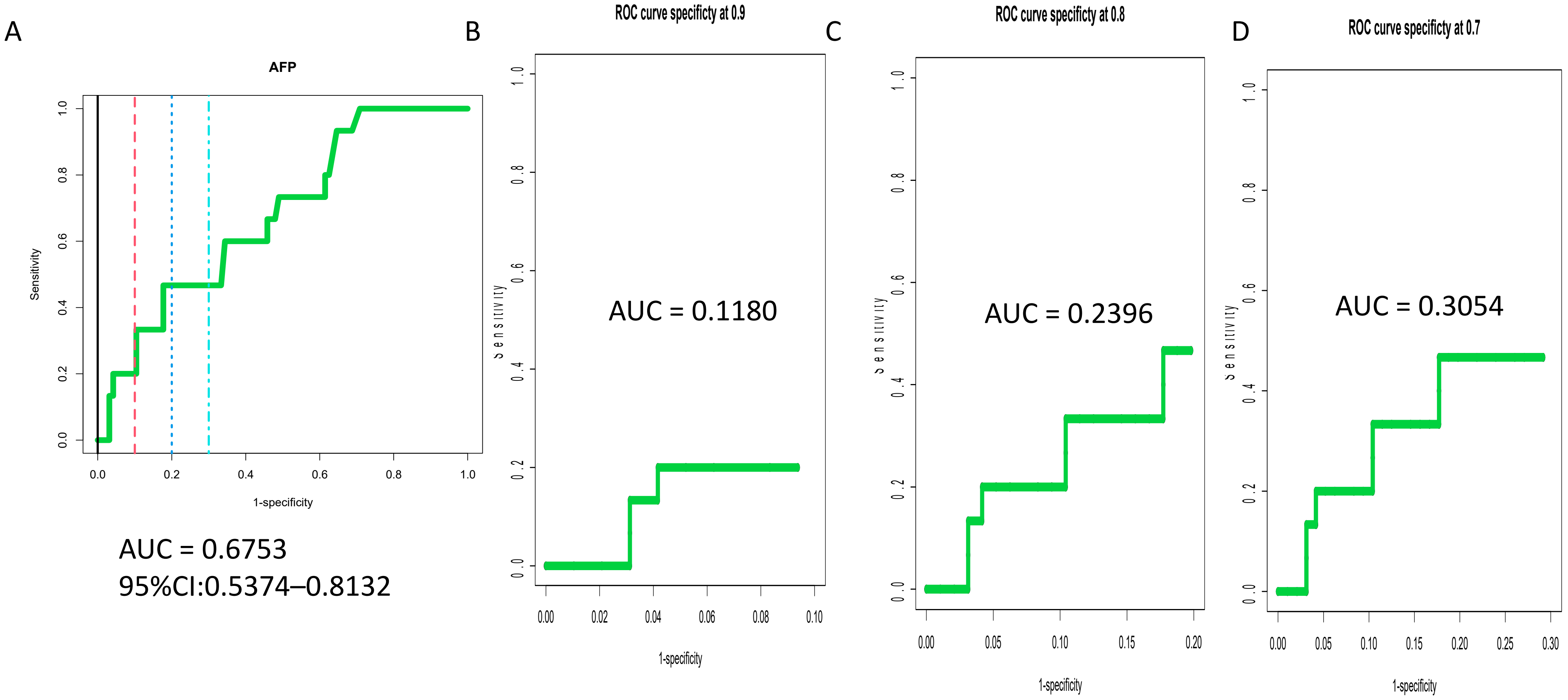

Supplementary Figure S9: AUROC of AFP in the UM cohort in patients at a time point of 7–12 months prior to HCC development. (A) Full ROC of Peg-IgG showing the specificity cut-offs at 90% specificity, 80% specificity and 70% specificity. Partial ROCS at 90% (B), 80% (C) or 70% specificity (D) are shown with the AUC for each partial ROC provided.

S10

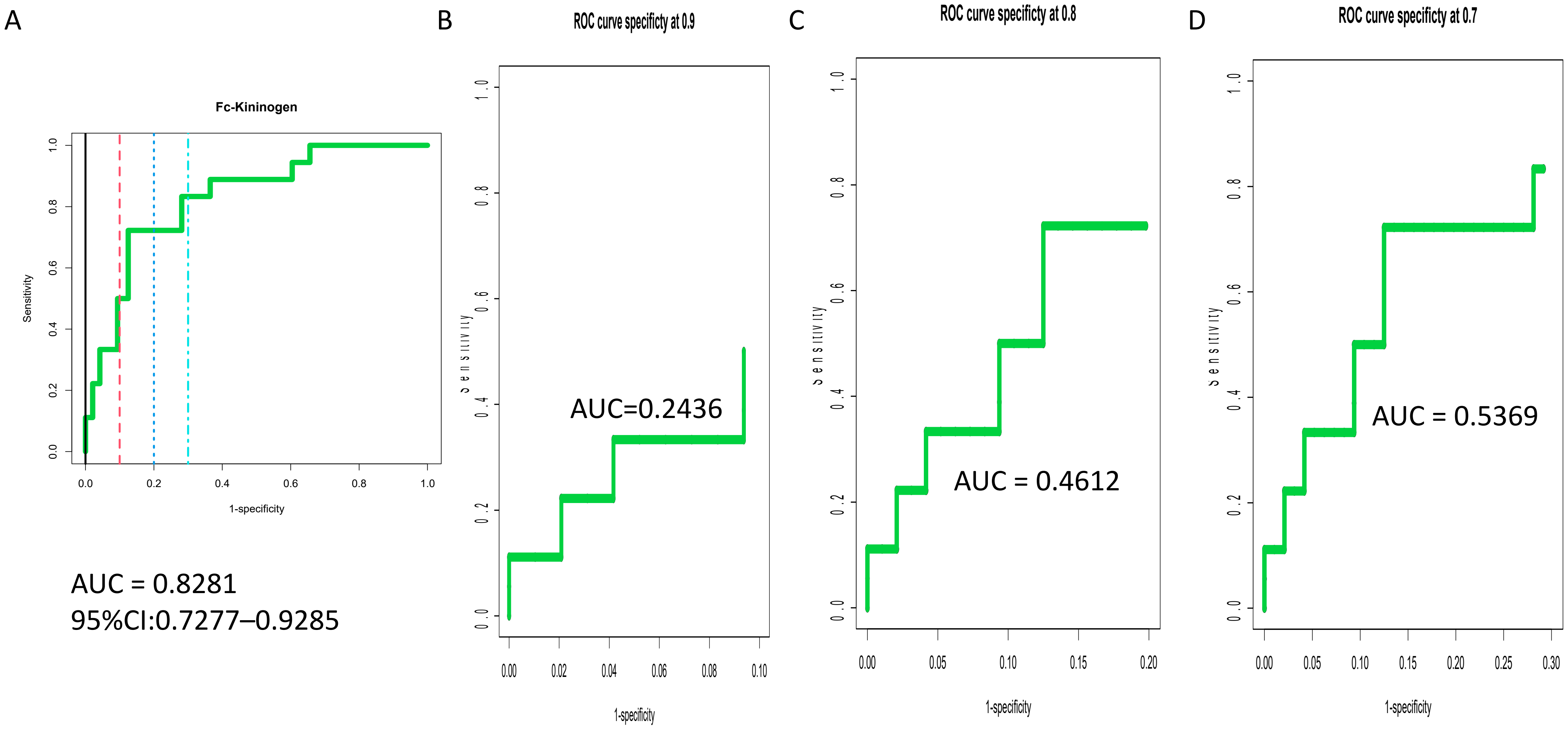

Supplementary Figure S10: AUROC of fucosylated kininogen in the UM cohort in patients at a time point of 1–6 months prior to HCC development. (A) Full ROC of Peg-IgG showing the specificity cut-offs at 90% specificity, 80% specificity and 70% specificity. Partial ROCS at 90% (B), 80% (C) or 70% specificity (D) are shown with the AUC for each partial ROC provided.

S11

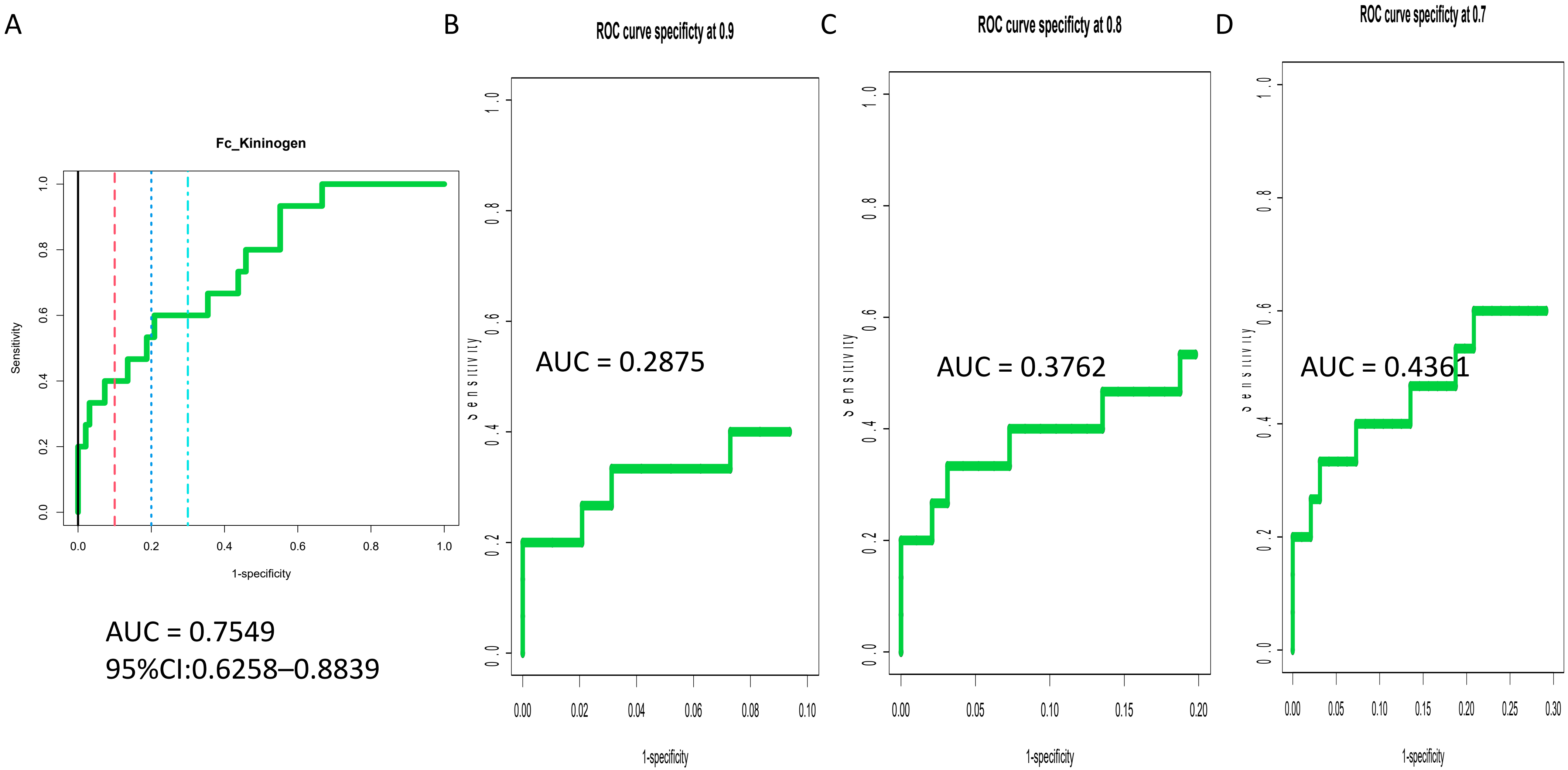

Supplementary Figure S11: AUROC of fucosylated kininogen in the UM cohort in patients at a time point of 7–12 months prior to HCC development. (A) Full ROC of Peg-IgG showing the specificity cut-offs at 90% specificity, 80% specificity and 70% specificity. Partial ROCS at 90% (B), 80% (C) or 70% specificity (D) are shown with the AUC for each partial ROC provided.

S12

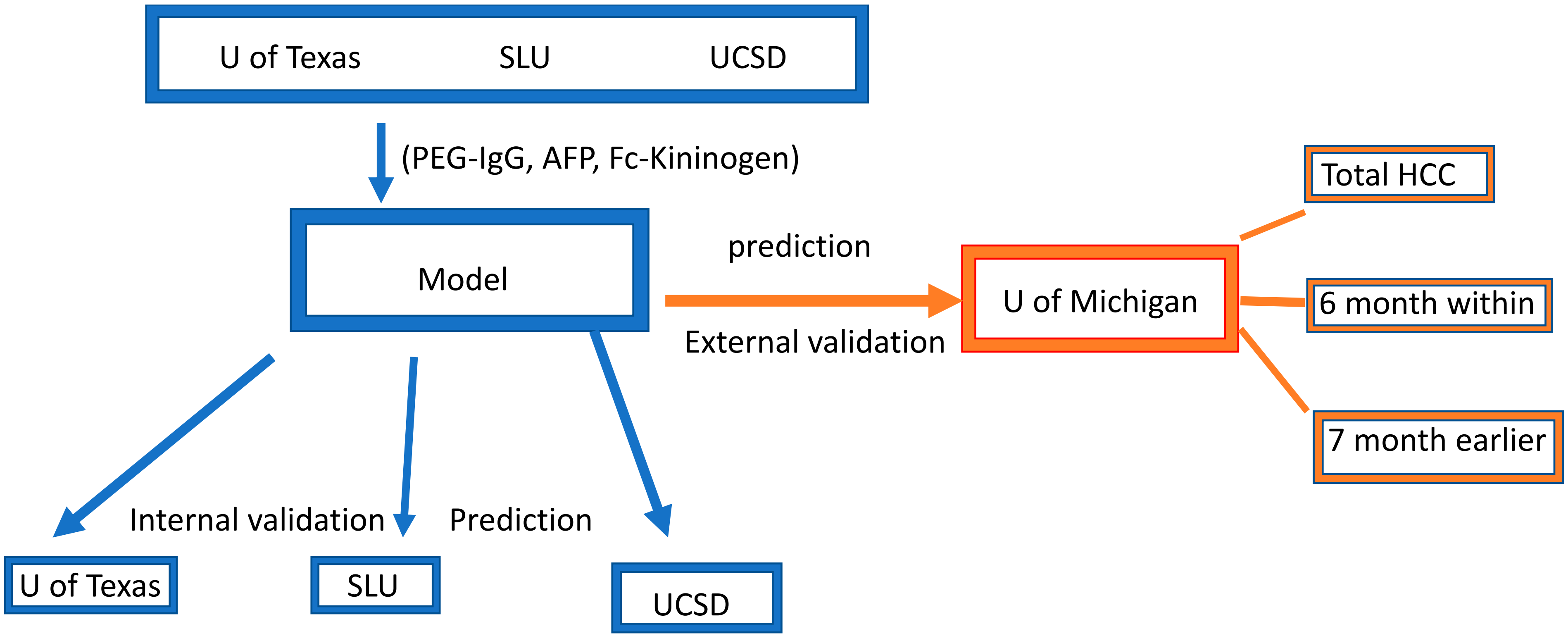

Normalizing PEG-IgG, AFP and Fc-Kininogen to unify 4 biomarkers of 4 cohorts to facilitate predictions

Model:

$$P(HCC)= \frac{1}{1+\exp(-[-076+(1.12*PEG\_IgG)+(1.09*logAFP)+(0.05*Fc\_Kininogen)]\,)}$$

Supplementary Figure S12: Workflow of model development. To improve the biomarker performance of PEG-IgG, this marker was incorporated into biomarker algorithm comprising AFP and fucosylated kininogen. Data from three “discovery” cohorts were pooled and used to develop a model for classification of HCC. The logistic regression model, as shown on the bottom, was composed of PEG-IgG, AFP, and fucosylated kininogen

S13

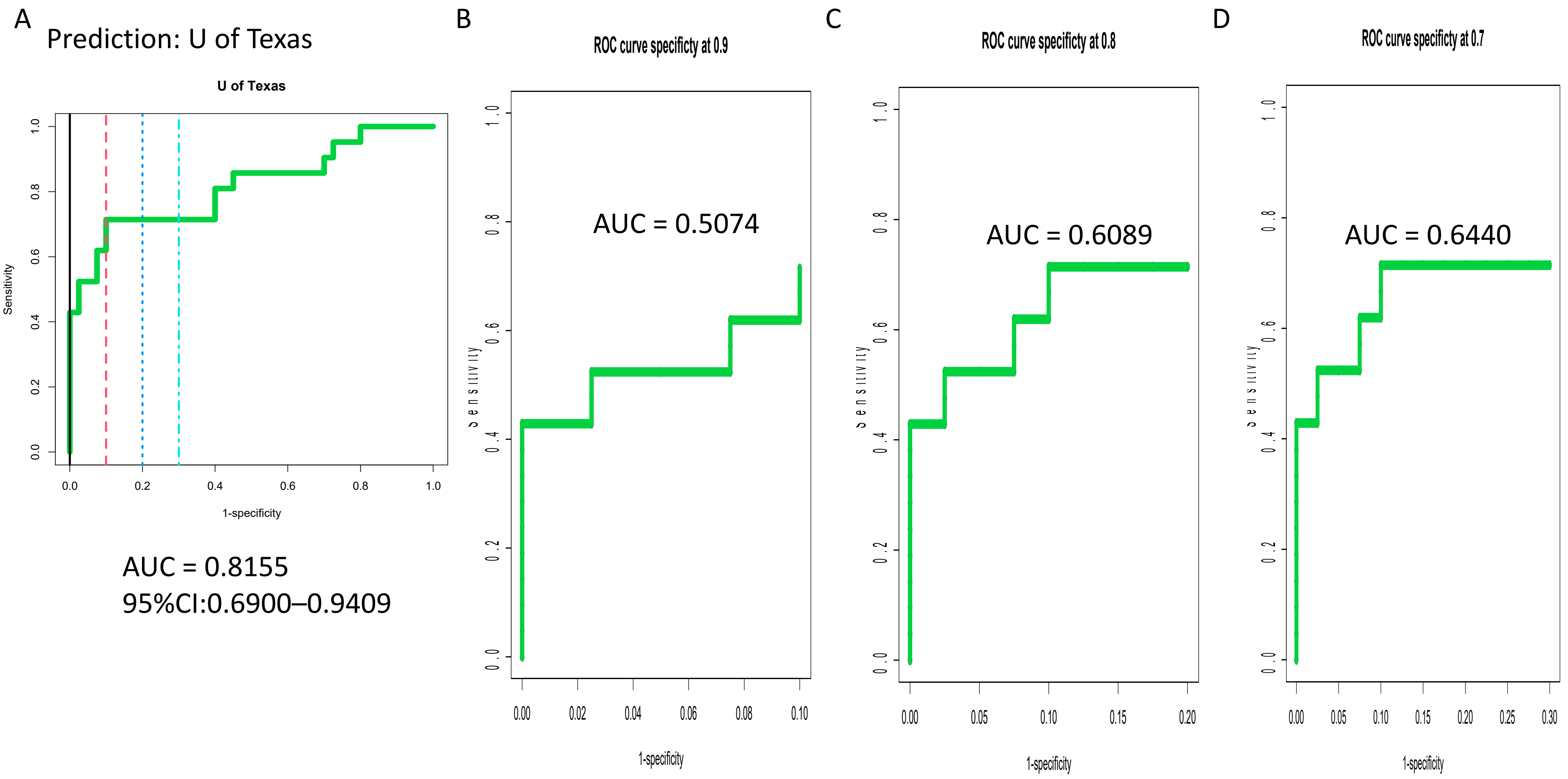

Supplementary Figure S13: AUROC of model in the UT cohort. (A) Full ROC of Peg-IgG showing the specificity cut-offs at 90% specificity, 80% specificity and 70% specificity. Partial ROCS at 90% (B), 80% (C) or 70% specificity (D) are shown with the AUC for each partial ROC provided.

S14

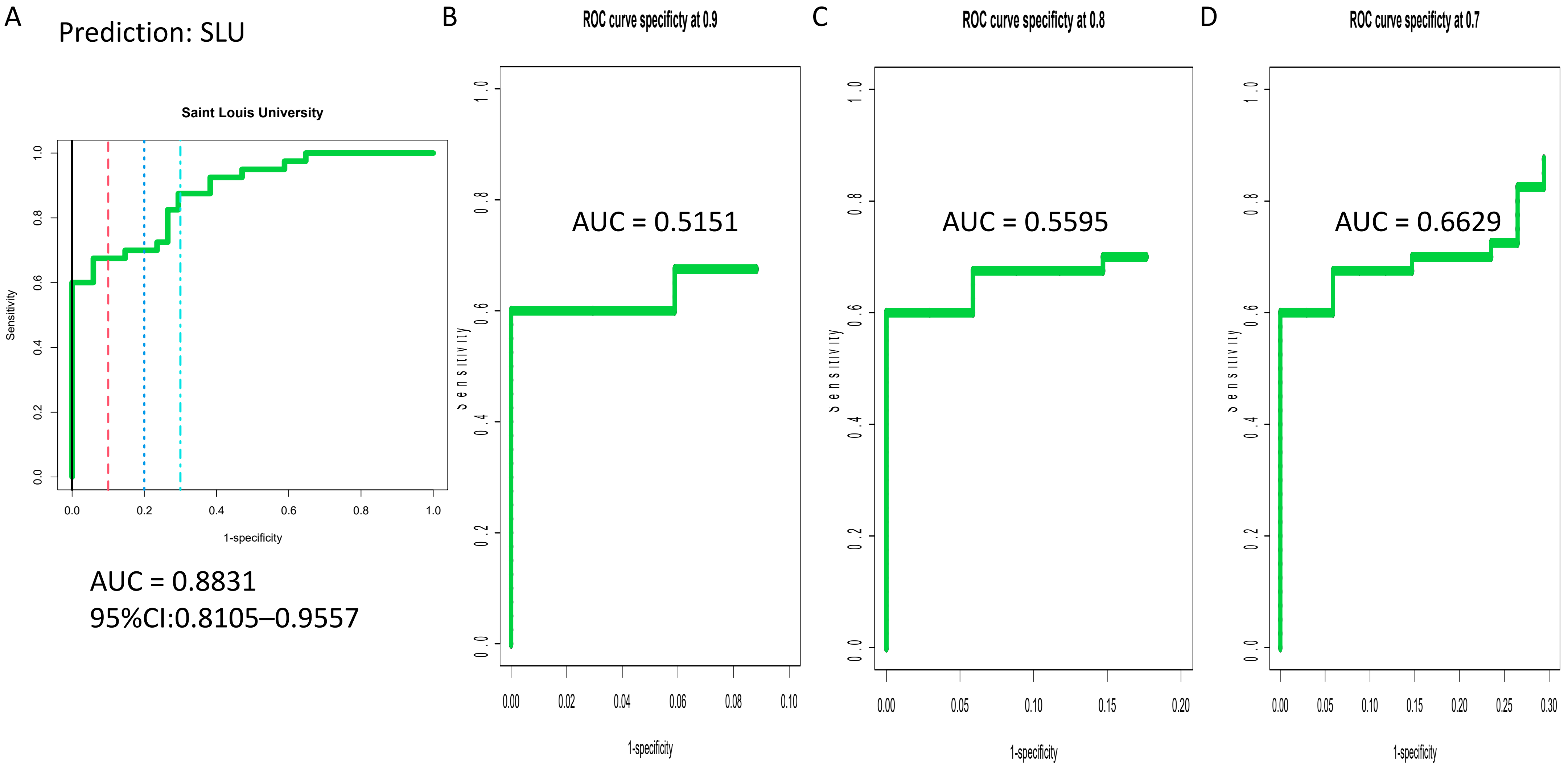

Supplementary Figure S14: AUROC of model in SLU cohort. (A) Full ROC of Peg-IgG showing the specificity cut-offs at 90% specificity, 80% specificity and 70% specificity. Partial ROCS at 90% (B), 80% (C) or 70% specificity (D) are shown with the AUC for each partial ROC provided.

S15

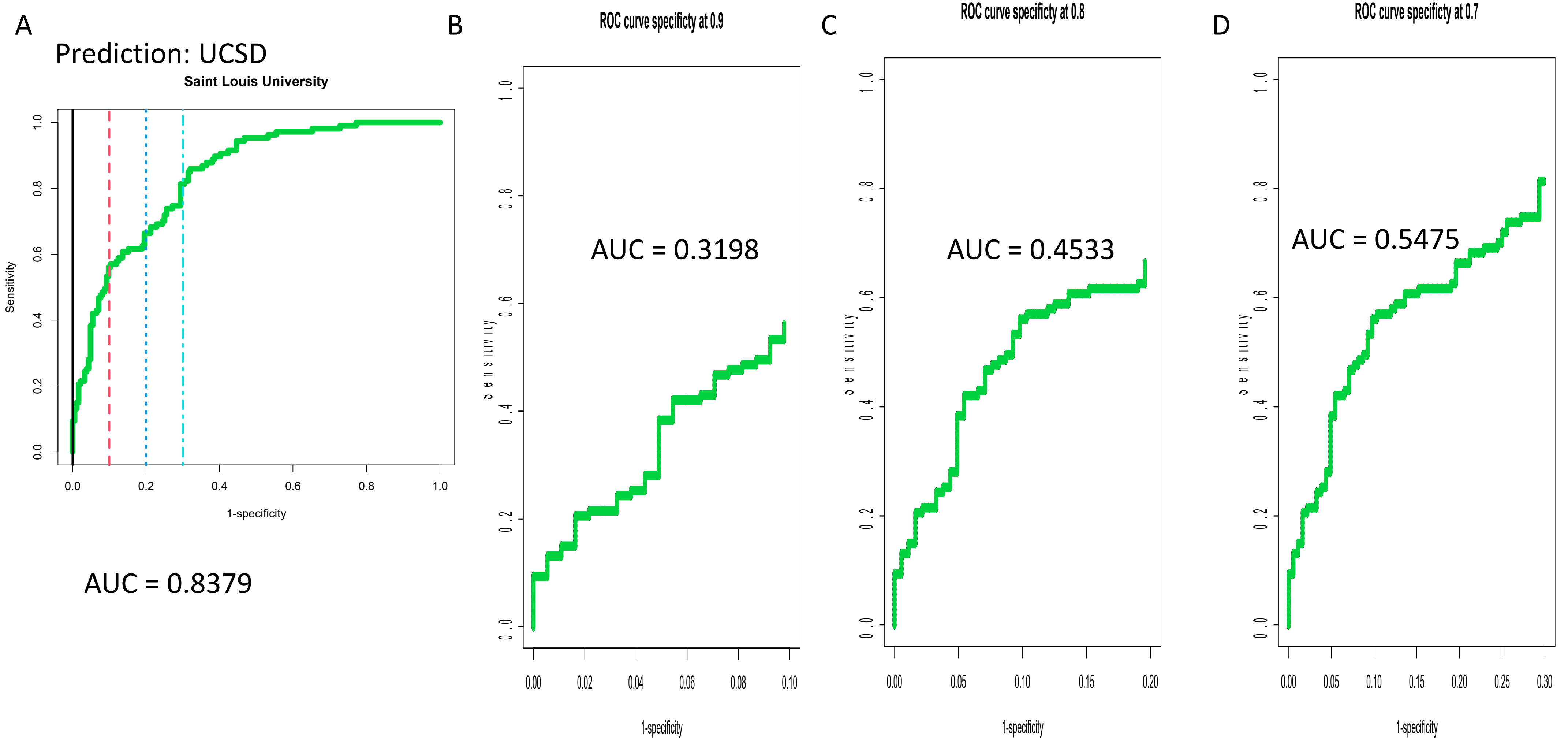

Supplementary Figure S15: AUROC of model in UCSD cohort. (A) Full ROC of Peg-IgG showing the specificity cut-offs at 90% specificity, 80% specificity and 70% specificity. Partial ROCS at 90% (B), 80% (C) or 70% specificity (D) are shown with the AUC for each partial ROC provided.

S16

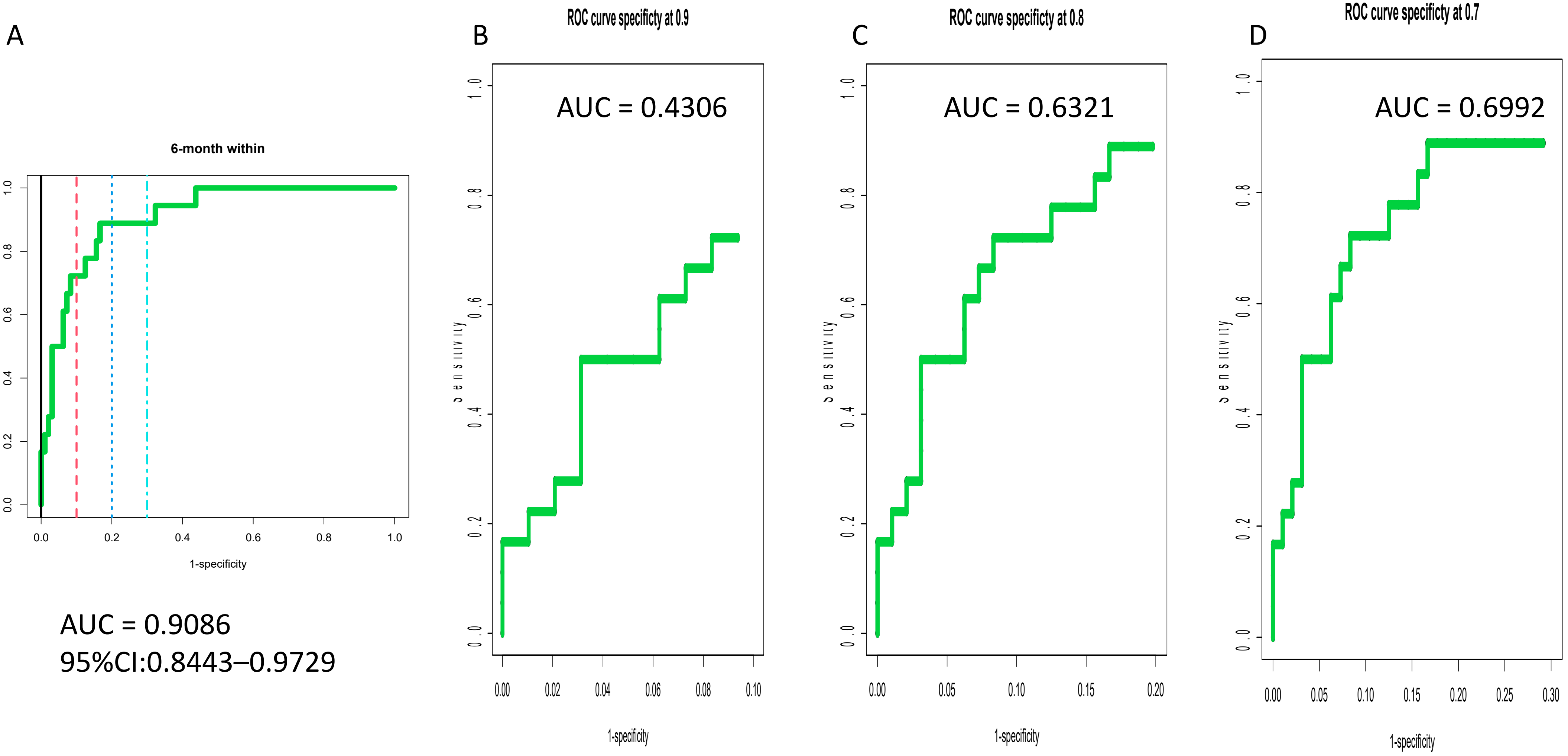

Supplementary Figure S16: External validation of model in the UM cohort in patients at a time point of 1–6 months prior to HCC development. (A) Full ROC of Peg-IgG showing the specificity cut-offs at 90% specificity, 80% specificity and 70% specificity. Partial ROCS at 90% (B), 80% (C) or 70% specificity (D) are shown with the AUC for each partial ROC provided.

S17

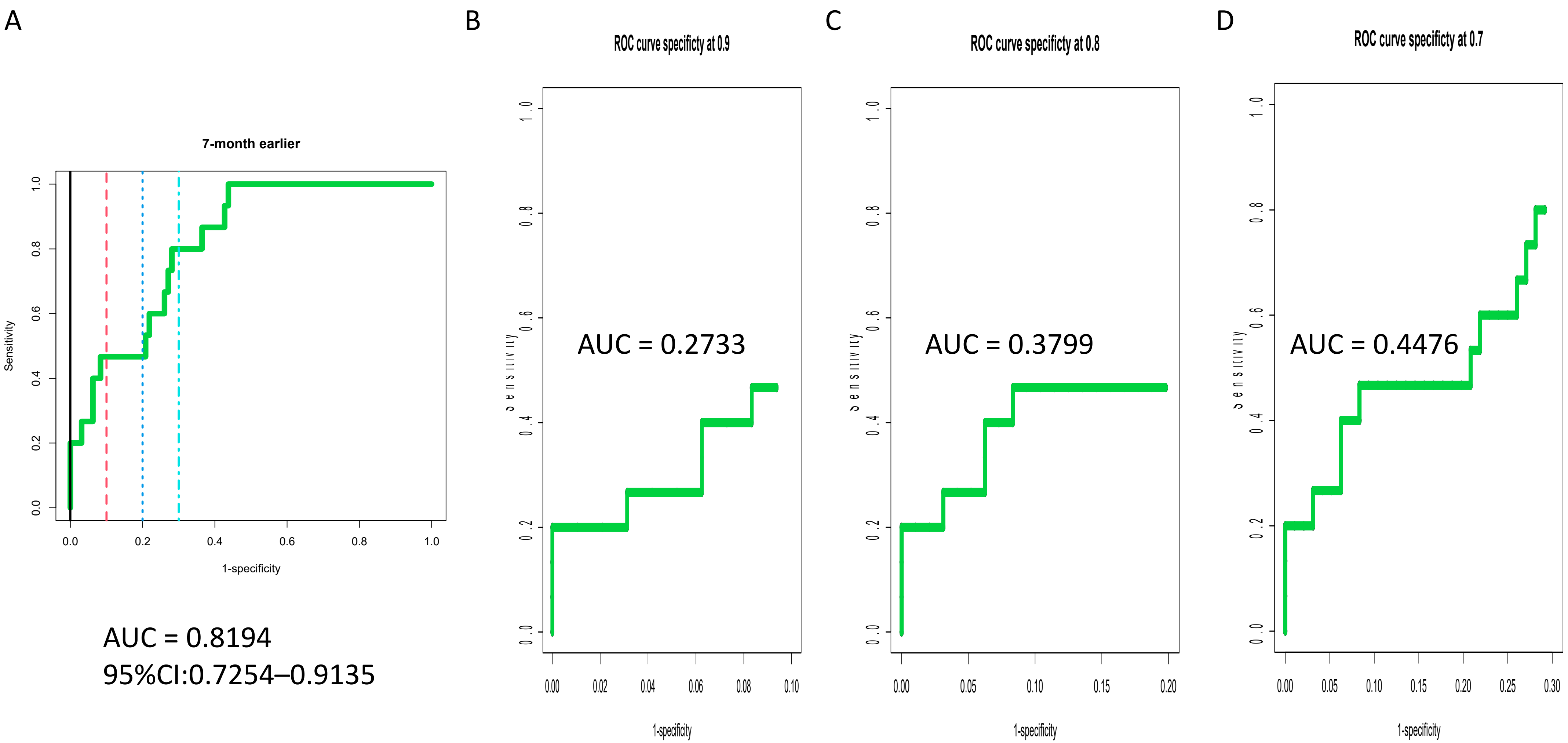

Supplementary Figure S17: External validation of model in the UM cohort in patients at a time point of 7+ months prior to HCC development. (A) Full ROC of Peg-IgG showing the specificity cut-offs at 90% specificity, 80% specificity and 70% specificity. Partial ROCS at 90% (B), 80% (C) or 70% specificity (D) are shown with the AUC for each partial ROC provided.

S18

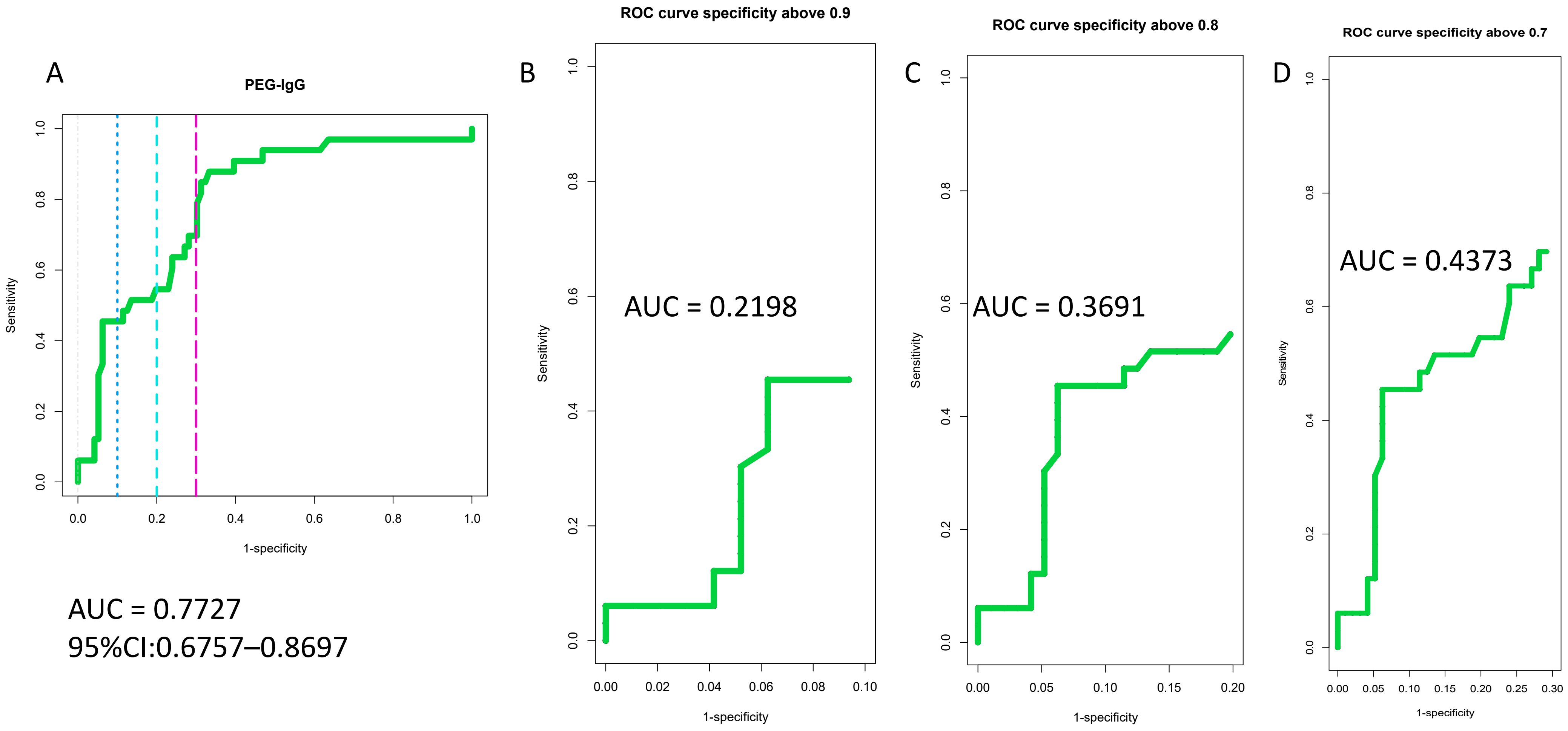

Supplementary Figure S18: AUROC of Peg-IgG in the UM cohort in patients with cancers falling within Milan criteria. (A) Full ROC of Peg-IgG showing the specificity cut-offs at 90% specificity, 80% specificity and 70% specificity. Partial ROCS at 90% (B), 80% (C) or 70% specificity (D) are shown with the AUC for each partial ROC provided.

S19

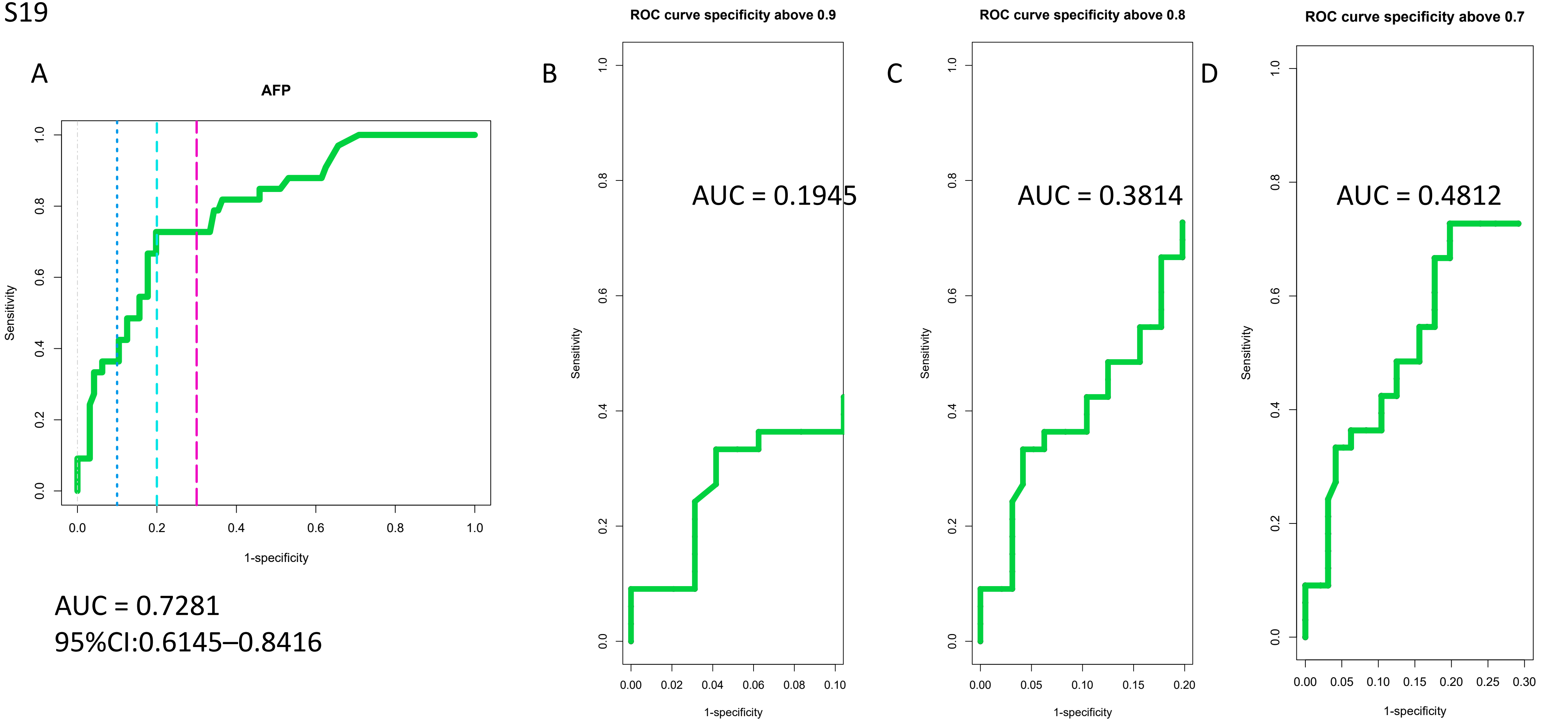

Supplementary Figure S19: AFP AUROC in the UM cohort in patients with cancers falling within Milan criteria. (A) Full ROC of Peg-IgG showing the specificity cut-offs at 90% specificity, 80% specificity and 70% specificity. Partial ROCS at 90% (B), 80% (C) or 70% specificity (D) are shown with the AUC for each partial ROC provided.

S20

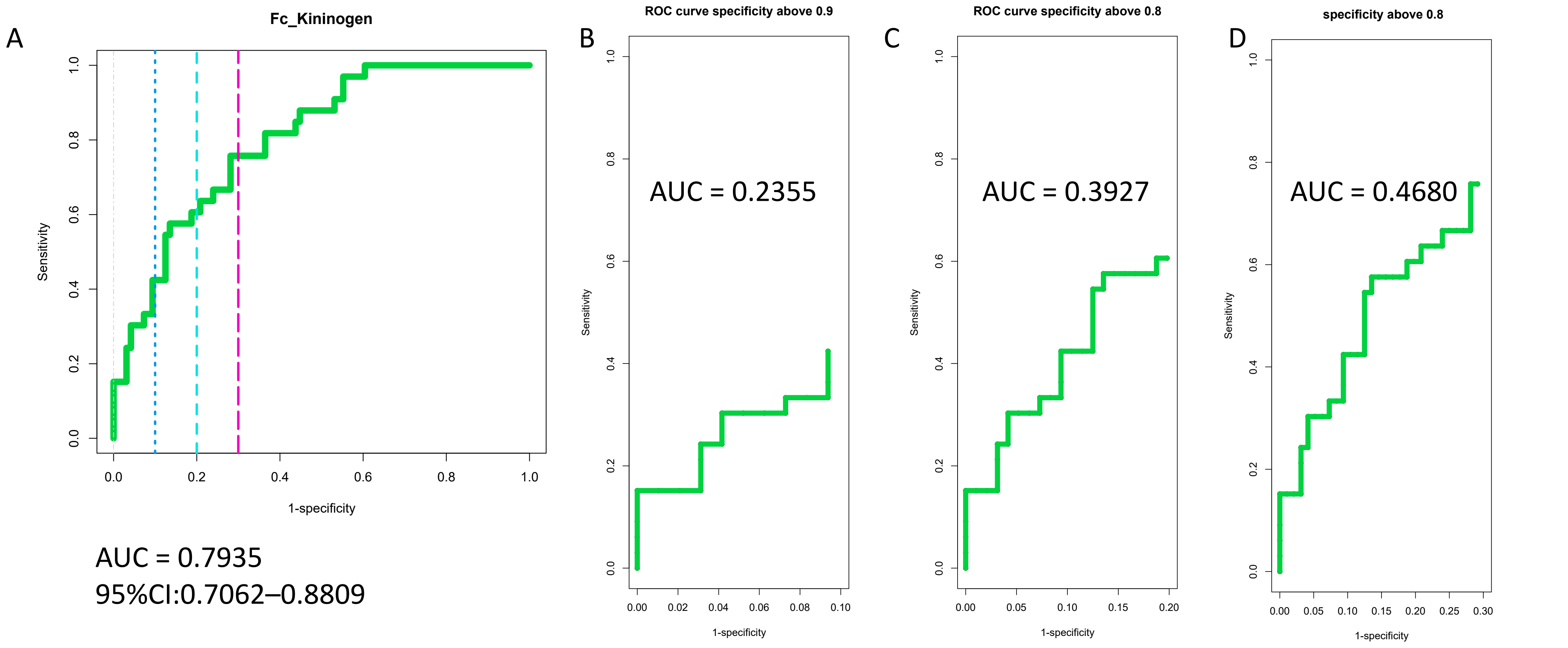

Supplementary Figure S20: Fucosylated kininogen AUROC in the UM cohort in patients with cancers falling within Milan criteria. (A) Full ROC of Peg-IgG showing the specificity cut-offs at 90% specificity, 80% specificity and 70% specificity. Partial ROCS at 90% (B), 80% (C) or 70% specificity (D) are shown with the AUC for each partial ROC provided.

Table S1. Comparison of previously published algorithms and the PEG-IgG related Algorithm

	**University of Texas**	**University of Michigan (all time points)**	**UCSD**
Sample	HCC:21, Cir:40	HCC:37, Cir:60	HCC:107, Cir:184
AFP	0.8810(0.7805–0.9814)	0.7768(0.6821–0.8715)	0.7267(0.6662-0.7872)
AFP,Age,Gender,ALK,AST (apparent validation) [1]	0.9524(0.8958–1)	0.8001(0.7093–0.8925)	0.8163(0.7670–0.8656)
AFP,Age,Gender,ALK,AST (predicted from published equation)[1]	0.9226(0.8558–0.9894)	0.7203(0.6186–0.8220)	0.7811(0.7266–0.8357)
AFP,Age,Gender,ALK,AST,Fc-kininogen(apparent validation) [2]	0.9738(0.9356–1)	0.8757(0.8011–0.9502)	0.8242(0.77565–0.8729)
AFP,Age,Gender,ALK,AST,Fc-kininogen (predicted from published equation)[2]	0.7310(0.5906–0.8713)	0.7811(0.6907–0.8714)	0.7442(0.6863–0.8022)
AFP, PEG-IgG, Fc-Kininogen (apparent validation)	0.9476(0.8909–1)	0.9383(0.8936–0.9839)	0.8406(0.7959–0.8853)
Note: AST and ALK are missing in cohort of Saint Louis University, so this cohort is not included in this comparison.			

1. Wang, M.; Devarajan, K.; Singal, A.G.; Marrero, J.A.; Dai, J.; Feng, Z.; Rinaudo, J.A.; Srivastava, S.; Evans, A.; Hann, H.W.; et al. The Doylestown Algorithm: A Test to Improve the Performance of AFP in the Detection of Hepatocellular Carcinoma. *Cancer Prev. Res.* **2016**, *9*, 172–179. https://doi.org/10.1158/1940-6207.CAPR-15-0186.
2. Wang, M.; Sanda, M.; Comunale, M.A.; Herrera, H.; Swindell, C.; Kono, Y.; Singal, A.G.; Marrero, J.A.; Block, T.; Goldman, R.; et al. Changes in the glycosylation of kininogen and the development of a kininogen based algorithm for the early detection of HCC. *Cancer Epidemiol. Biomark. Prev.* **2017**, *26*, 795–803. https://doi.org/10.1158/1055-9965.EPI-16-0974.
